# Computer Aided Written Character Feature Extraction in Progressive Supranuclear Palsy and Parkinson’s Disease

**DOI:** 10.3390/s22041688

**Published:** 2022-02-21

**Authors:** Paula Stępień, Jacek Kawa, Emilia J. Sitek, Dariusz Wieczorek, Rafał Sikorski, Magda Dąbrowska, Jarosław Sławek, Ewa Pietka

**Affiliations:** 1 Faculty of Biomedical Engineering, Silesian University of Technology, 41-800 Zabrze, Poland; paula.maria.stepien@gmail.com (P.S.); ewa.pietka@polsl.pl (E.P.); 2 Division of Neurological and Psychiatric Nursing, Faculty of Health Sciences, Medical University of Gdansk, 80-211 Gdansk, Poland; emilia.sitek@gumed.edu.pl (E.J.S.); jaroslaw.slawek@gumed.edu.pl (J.S.); 3 Department of Neurology, St. Adalbert Hospital, Copernicus PL Ltd., 80-462 Gdansk, Poland; magdakobierowska@o2.pl; 4 Department of Rehabilitation, Faculty of Health Sciences, Medical University of Gdansk, 80-219 Gdansk, Poland; dariusz.wieczorek@gumed.edu.pl; 5 Department of Rehabilitation, Saint Vincent a Paulo Hospital, Pomeranian Hospitals Ltd., 81-519 Gdynia, Poland; sikorski@gumed.edu.pl

**Keywords:** baseline estimation, character recognition, computer aided diagnosis, neurodegenerative diseases, pattern analysis, writing analysis

## Abstract

Parkinson’s disease (PD) and progressive supranuclear palsy (PSP) are neurodegenerative movement disorders associated with cognitive dysfunction. The Luria’s Alternating Series Test (LAST) is a clinical tool sensitive to both graphomotor problems and perseverative tendencies that may suggest the dysfunction of prefrontal and/or frontostriatal areas and may be used in PD and PSP assessment. It requires the participant to draw a series of alternating triangles and rectangles. In the study, two clinical groups—51 patients with PD and 22 patients with PSP—were compared to 32 neurologically intact seniors. Participants underwent neuropsychological assessment. The LAST was administered in a paper and pencil version, then scanned and preprocessed. The series was automatically divided into characters, and the shapes were recognized as rectangles or triangles. In the feature extraction step, each rectangle and triangle was regarded both as an image and a two-dimensional signal, separately and as a part of the series. Standard and novel features were extracted and normalized using characters written by the examiner. Out of 71 proposed features, 51 differentiated the groups (*p* < 0.05). A classifier showed an accuracy of 70.5% for distinguishing three groups.

## 1. Introduction

According to the American National Institute of Neurological Diseases and Stroke, there are over 600 neurological disorders. The differential diagnosis of most neurodegenerative disorders in clinical practice relies heavily on the clinician’s experience and short screening measures feasible in busy movement disorders and/or dementia clinic.

The diagnosis of most sporadic movement disorders of neurodegenerative etiology remains a clinical challenge as many motor features lack specificity and a full neuropsychological workup is not always available. Even in the era of sophisticated technology enabling the tracking of subtle motor features, a clinical neurological examination is the core element of the diagnostic process [1]. Quantitative analysis of data gathered during routine clinical assessment could be a valuable add-on to the qualitative interpretation based on the clinical judgment. Tools allowing qualitative and quantitative approaches to interpretation are particularly approachable for a busy clinician.

Parkinson’s disease (PD) is one of the most common neurodegenerative diseases as it affects up to 1.5% of the population over 65 years of age [2]. PD is characterized mainly by motor symptoms such as bradykinesia, accompanied by postural instability, muscle rigidity, and/or rest tremor. However, currently, non-motor symptoms, covering a broad range of areas, including, for example, gastrointestinal, sleep, urinary, sexual, and cognitive and behavioral changes, are being increasingly recognized [3]. Although PD is an incurable disease, its correct and rapid diagnosis is indispensable, as disease symptoms are responsive to pharmacological and surgical interventions.

Progressive supranuclear palsy (PSP), also known as Steele–Richardson–Olszewski disease (often classified as an atypical parkinsonian syndrome), is a tauopathy characterized by vertical gaze palsy, early falls, and executive deficits. PSP occurs in about 7 out of 100 thousand persons. The average age of onset is 63 years, whereas the average survival time is 5–7 years. Behavioral and cognitive manifestations occur in the first year of the disease in 52% of patients. At the advanced stage, this number increases to over 80%. In a majority of cases, the severity of cognitive and behavioral symptoms corresponds to the clinical criteria of behavioral variant frontotemporal dementia [4]. Cognitive impairment manifests itself mainly in the form of executive dysfunction [5]. Executive deficits are more severe than in PD [6] and usually are one of the core features [7]. Those deficits’ characteristic pattern includes inhibition difficulties (impulsivity) and perseveration (inability to change mental set leading to behavior repetition). Those distinctive executive deficits are accompanied in PSP by other executive problems that are present as well in PD, such as initiation problems.

Parkinson’s disease (PD) and progressive supranuclear palsy (PSP) share many motor features (e.g., rigidity) and non-motor symptoms (e.g., apathy) [8]. Differentiating PD from PSP is not an easy clinical task and requires not only neuroradiological [3] and neuropsychological assessment but also patient follow-up and assessing response to medication [3].

The differences in symptoms between PSP and PD are known under the acronym FIGS [9]: frequent sudden falls, ineffective medication, gaze palsy, and speech and swallowing changes.

Symptoms consistent with FIGS seem relatively easy to detect using telemedicine systems. Fall control requires a relatively complex system equipped with inertial sensors, cameras, or diaries [10] (useful also in determining the impact of medication on daily functioning). Research on the speech changes registered using microphones involves both tracking the disease progression and attempting to determine a vector of features allowing parkinsonian syndromes to be distinguished from one another [11,12]. Tracking eye movements also seems promising, taking into account both the number of studies being conducted and patents developed [13,14,15].

Most advanced computational analyses used for either diagnostic purposes or tracking the disease progression focus on either motor or speech abnormalities [16,17]. They require specialized equipment at the time of data acquisition and data processing. Of note, writing and drawing tasks that are easily administered during routine clinical visits can shed light on both motor and cognitive function [18]. As kinematic aspects of handwriting movements are dependent on dopaminergic transmission [19], graphomotor tasks may be susceptible to subtle motor abnormalities such as emergent micrographia [20]. Writing tasks can uncover not only language but also visuospatial or executive problems (e.g., dysexecutive agraphia [19]). Copying alternating designs may reveal, among others, motor problems [21]. Those tasks offer not only the possibility of qualitative analysis at the patient’s bedside, but they can also be further quantitatively analyzed to detect subtle differentiating features or track changes over time.

The Luria’s Alternating Series Test (LAST) is a known clinical tool to detect perseveration. It requires the completion of a series presented by the examiner, consisting of alternating rectangles and triangles. Set-shifting problems characteristic for prefrontal and/or frontostriatal dysfunction manifest in LAST as the continuous [22] drawing of the same element (perseveration) [23].

The concept of perseveration is essential in the LAST test. It is defined as the inability to change the thinking or response to a stimulus due to the ”failure of the automatic adjustment mechanism” [24]. Tools for detecting defects in self-regulation skills are usually very sophisticated and education-dependent [25] and thus not feasible in a busy movement disorders clinic. As perseveration errors are unequivocal markers of executive deficits, most short executive tests include items focusing on the detection of perseveration [26]. In addition to the presence of perseveration in the LAST, the number of errors in the series (corrected and uncorrected) and the proportion of uncorrected errors can be used as an indicator of the severity of set-shifting problems. By referring this to the number of all characters in the series, one can get the percentage and normalize the result [27].

The LAST has already been used in studies focusing on neurodegenerative diseases. In their work, Nomm et al. [28] extracted features such as velocity, acceleration, jerk, number of strokes, pressure, and horizontal and vertical components by using this method. The introduced method consists of four tasks: (1) continuing, (2) copying, and (3) tracing a pattern, and (4) copying a sinusoidal line on a tablet using a stylus. The test is carried out using a tablet, and thus in the setting that do not correspond to the original assumptions of the test. Furthermore, only the first task is consistent with the original paper version of the test, yet the dimensions of the screen are not suitable. The differences between the patterns are calculated using the Dynamic Time Warping algorithm [29] for the entire waveform. Interestingly, only kinematic and no dimensional features are taken into account.

The employment of kinematic features is present in many recent papers on handwriting analysis [30,31]. However, despite all the recent work on LAST, one has to bear in mind that the dynamic approach can only be applied to the recent data, whereas over the past 60 years, the Luria test data were traditionally collected using a sheet of paper and a pencil. The decades of archived cases combined with the patient’s record may elucidate the interaction of cognitive and motor features in movement disorders. Moreover, the paper version is still in use, as very few neuropsychologists or neurologists own digitizers or are even familiar with such equipment. This study is therefore concerned with the classical paper version of LAST.

The goal is to automatically process the test and extract features for the analysis and quantitative evaluation. This study is intended as a first step towards the automated analysis of contemporary as well as archived data and, in the long term, towards a computer aided diagnosis system.

In our previous work [32], we already explored the basic differences between the characters drawn by an examiner and examinee in the PSP group. The presented work aims to introduce and to select features that are able to differentiate three groups: Parkinson’s disease (PD), progressive supranuclear palsy (PSP), and seniors with no neurological disorders (CON).

This approach uses image and signal analysis techniques to automatically extract features measured until now with a ruler or not considered at all. A simple paper-and-pencil task that is usually interpreted only qualitatively can also provide quantitative information useful for differential diagnosis.

All data forming part of a routine examination were anonymized and provided along with selected demographic data. The study was approved by the Independent Bioethics Committee for Scientific Research at the Medical University of Gdańsk, Poland (pinkapproval number NKBBN/501/2013 from 14 January 2014).

### Contribution

The main contribution of this work is as follows:

- In clinical practice, the LAST has been so far evaluated only on the basis of observations and manually performed, simplified measurements. In our approach, the analysis is fully automatic. The results are compared with expert performances.
- In prior studies, a traditional sheet of paper and a pencil were replaced with a tablet and stylus, yielding a set of dynamic features and increasing the amount of information extracted from handwriting. In this study, a first fully automatic approach to the traditional paper–pencil LAST is introduced. No dynamic features are extracted, however.
- The baseline of the LAST series is calculated using the BEADS (Bias Elimination Algorithm for Deep Sequencing) algorithm—a recent baseline estimation approach—which has (to date) not been used in the area of computer-aided diagnosis of neurodegenerative diseases.
- In contrast to the previous approaches, in this study, the characters in the LAST series are analyzed separately and not only as one continuous drawing.
- The *NW coefficient* (an index based on the Needelman–Wunsch algorithm) is introduced to evaluate the correctness of the character order in the series. It can be applied to both automatic and manual evaluation.

## 2. Methods

Luria’s Alternating Series Test is part of a comprehensive neuropsychological assessment administered by a neuropsychologist. The patient sits at the desk with their dominant hand and the elbow positioned comfortably on the desk surface. If necessary, eyeglasses are used for vision correction. The test consists of two steps: (1) the examiner presents a short sequence of alternating connected shapes (open triangles and rectangles) drawn in front of the patient on a sheet of paper using a pen, (2) the examinee continues the pattern using the pencil until the end of the page, if possible without lifting the pencil. In this study, the acquired paper versions are processed to automatically extract features allowing the indication of the patient’s neurological state.

**Figure 1:**
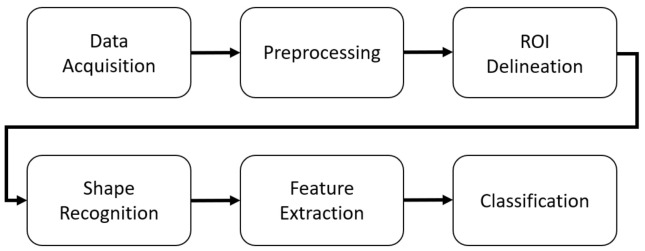
Workflow.

The approach consists of several steps (Figure 1). The acquired data are preprocessed. The regions of interest (ROIs) are delineated, and the characters are recognized as rectangles or triangles. Features are extracted, and the feature vector is used in the final step as the input of an SVM classifier that assigns the patients into one of three groups: Parkinson’s disease (PD), progressive supranuclear palsy (PSP), and neurologically intact (CON).

### 2.1. Data Acquisition

**Figure 2:**
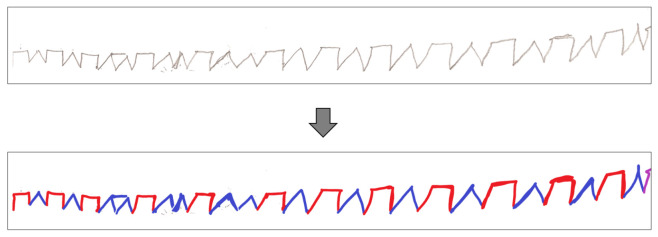
Expert labeling (ground truth pattern).

The paper versions of the test are digitized using an HP Deskjet Ink Advantage K209a scanner at 600 dpi and stored as 8-bit grayscale, uncompressed TIFF (Tagged Image File Format) or PNG (Portable Network Graphics) files. The data are transferred into the MATLAB environment. In order to detect the ground truth pattern for evaluation (discussed in Section 3), after the acquisition, each character is manually labeled as a rectangle (red) or triangle (blue) using a simple raster graphics editor (Figure 2). The first five characters drawn by the expert are tagged separately to be used for normalization in the future steps.

### 2.2. Preprocessing

The suppression of artifacts resulting from the paper’s uneven texture or the scanner limitations is obtained by the background removal. As the image features a white background (typically well over 90% of the scan area), the tallest high-intensity histogram waveform indicates the threshold for binarization. The obtained image is subjected to morphological opening using a symmetrical structural element in order to reduce scanning artifacts enhanced in binarization.

Next, skeletonization employing the Lam algorithm [33] is performed on the LAST image form. The result is resampled (left-to-right), and a single y-value is chosen for each x-value, defining a signal-like representation of LAST. It is used, along the image form, in selected steps described later, including the separation of characters.

In order to find the moment of transition between two adjacent characters, the aligned signal form of LAST is required. It is calculated from the signal form using the BEADS (Bias Elimination Algorithm for Deep Sequencing) algorithm [34] originally designed to remove baseline, background, or drift and random noise from sparse signals in analytical chemistry, yet recently used, e.g., in movement tracking [35].

In the BEADS approach, the signal y is considered to be composed of a baseline f, peaks x, and stationary white Gaussian noise w. During the optimization procedure, the components can be retrieved [34]:

(1) $$\hat{x}={arg min}_{x}\left\{F\left(x\right)=\frac{1}{2}{\left||H\left(y-x\right)|\right|}_{2}^{2}+\lambda_{0}\sum_{n=0}^{N-1}\theta_{\epsilon }\left(x_{n},r\right)+\sum_{i=1}^{M}\lambda_{i}\sum_{n=0}^{N_{i}-1}\phi \left({\left[D_{i}x\right]}_{n}\right)\right\},$$

where H is high-pass filter matching the low-pass filter L given as an argument, $D_{i}$ denotes the difference operator of the order *i*, while $\theta_{\epsilon }$ nand ϕ are continuous and differentiable penalty functions promoting the sparsity of *N-dimensional* signal and peak derivatives. The sparsity of a signal and its first and higher derivatives are weighted by $\lambda_{0}$, $\lambda_{1}$, $\lambda_{2}$,…, $\lambda_{M}$ input parameters.

**Figure 3:**
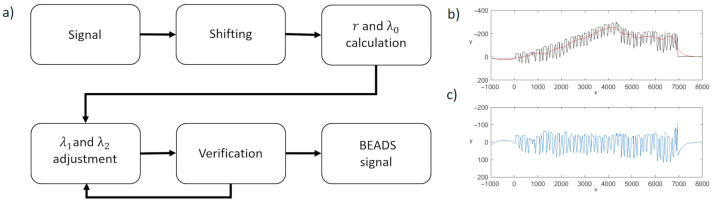
(**a**) Baseline estimation algorithm. (**b**) The processed signal (black) and the calculated baseline (red). (**c**) The signal after baseline subtraction.

In the paper, the penalty functions $\theta_{\epsilon }\left(x,r\right)$ and ϕ proposed in [34] are used. The low-pass filter with a cut-off frequency of $10^{-4}$ Hz (selected experimentally) is employed. Based on the outcome of the experiments on the signal samples originating from the control group, an initial value of $\lambda_{0}$ is set to the inverse of the of signal standard deviation, whereas $\lambda_{1}=\lambda_{2}=2\cdot \lambda_{0}$ and *r* are set to the inverse of the signal variance. The $\lambda_{1}$ and $\lambda_{2}$ are subsequently adjusted based on the signal characteristics by iterative application of BEADS. First, the signal is shifted so that its first value is zero ($y_{1}=0$). Next, BEADS is applied. If the calculated maximum of the obtained baseline exceed the values of the original signal significantly, one of the parameters is doubled, and the baseline is calculated again (Figure 3a). In the final step, the calculated baseline (Figure 3b) is subtracted from the original signal, and the desired *aligned* signal is obtained (Figure 3c).

### 2.3. Character Separations (ROI Delineation)

**Figure 4:**
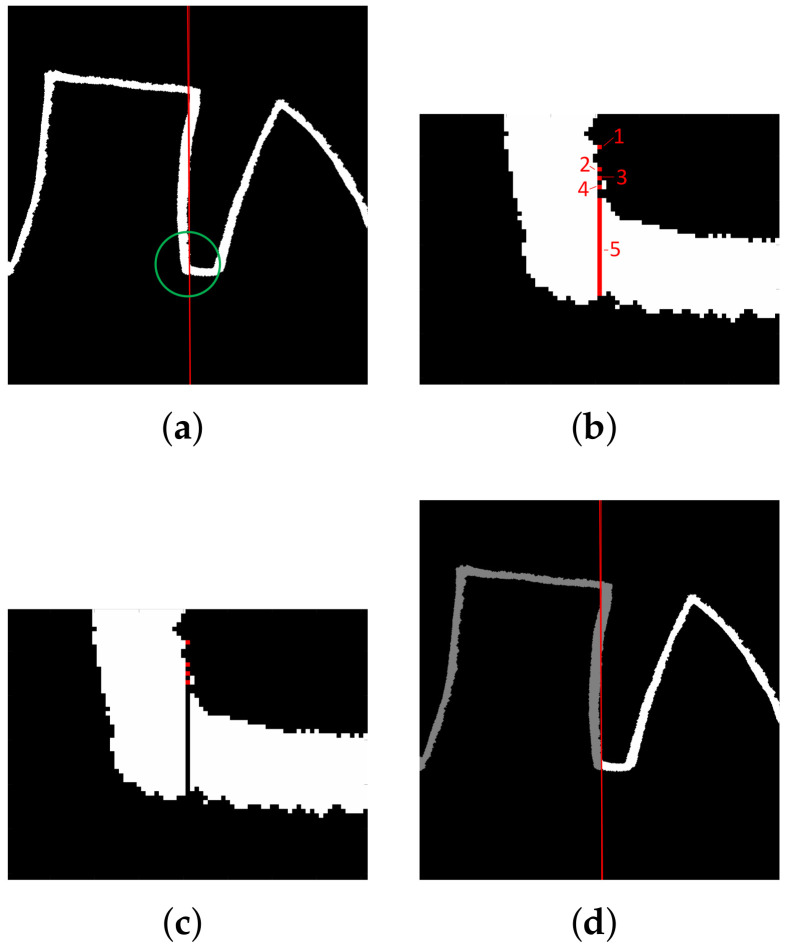
(**a**) Calculated location of separating column. (**b**) Candidate components. (**c**) Separating component no. 5 removed. (**d**) Obtained shapes marked with different shades.

The minimum of the signal located close to the baseline marks the column of the image in which the transition between two adjacent characters takes place. The coordinates indicate the image section separating characters in the image form of the LAST (Figure 4a–c). The labeling-based procedure (Figure 4d) separates shapes: connected components, including askew fragments, are extracted, and consecutive labels denote the following shapes.

As a result, the characters of the image and signal form of the LAST are separated. In the image, the characters define sub-images (regions of interest (ROIs)), whereas in the signal, continuous groups of samples are obtained.

### 2.4. Character Recognition

Once the characters are separated, they are classified either as a rectangle or triangle class. Initially, typical shapes are selected. These shapes are used as models in the Dynamic Time Warping (DTW) algorithm [36] operating on the signal form of the LAST. On the basis of the minimal distance from models, the remaining characters are assessed.

The DTW is typically used to align similar sequences varying in speed. Time warping permits several samples from a first sequence to be matched with a single sample from a second sequence and the other way around, compensating for different sampling or speed. Moreover,

- First and last indices of both sequences are always matched. However, they may be additionally matched to some other samples as well;
- The mapping must be monotonically increasing (samples cannot be reorganized);
- Every sample must be matched (samples cannot be omitted);
- Matching yielding smallest distance is selected in an iterative procedure involving comparison of every sample of both sequences.

The cumulative distance (e.g., Euclidean or squared metric) of matched samples determines *DTW distance*. A zero DTW distance denotes a perfect match.

In the paper, DTW is used in a constrained version: a mapped sample cannot be matched with a sample outside the sliding window with a width set to 5% of the longer sequence. The models and examined characters are comparable in size and normalized. The Euclidean metric is used.

The typical shapes (models) are obtained primarily from the image representation of the currently processed LAST:

- In the image, if the ratio of the area of the smallest triangle *T* circumscribing the character and the sum of the areas of the smallest rectangle *R* and the smallest triangle circumscribing the character (R+T) is smaller than 0.55, then the character is considered as a triangle model;
- In the image, the examined character may be considered a rectangle (triangle) model if (1) the area of the minimum enclosing rectangle (triangle) is smaller than the area of the minimum enclosing triangle (rectangle) and (2) the corresponding *IF rectangle* (*IF triangle*) ratio is smaller than 0.55 (see Table A1 in Appendix A for definitions and the methods). The 0.55 threshold was selected experimentally based on 10 individual examined shapes of each kind to allow for slightly deformed models and reject significantly malformed shapes.
- In the image, if the character contains a horizontal line longer than 0.25 of the total character width according to the Hough transform [37], then the character is considered as a rectangle model (the 0.25 threshold was selected experimentally based on the analysis of the ten individual shapes of each kind to allow for regular and slightly deformed templates).

**Figure 5:**
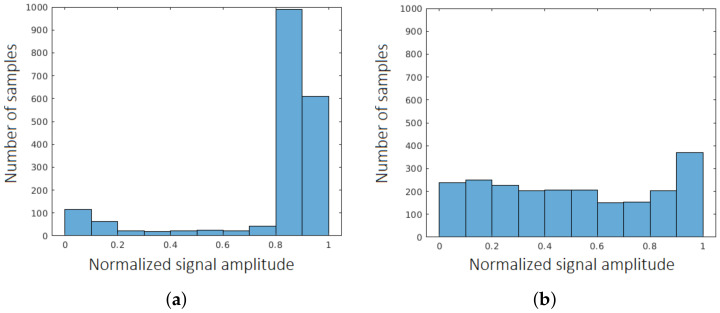
Histogram of the number of samples and their normalized amplitude for an exemplary (**a**) rectangle and (**b**) triangle.

Moreover, some templates are extracted from the signal itself, based on the amplitude analysis:

- In the normalized signal, the ratio of the number of the samples with the value (amplitude) higher than the 80% of the maximum value of the signal (Figure 5) and the total number of samples exceeds 66% then the character is considered as a rectangle model, whereas if it is lower than 33%, then the character is considered triangle model (cf. Histogram feature definition and comment in Table A2, Appendix A);

Three artificial models are employed if no characters of a given type are selected: a perfect rectangle and two isosceles trapezoids or a perfect triangle and two mirrored, right rectangles.

**Figure 6:**
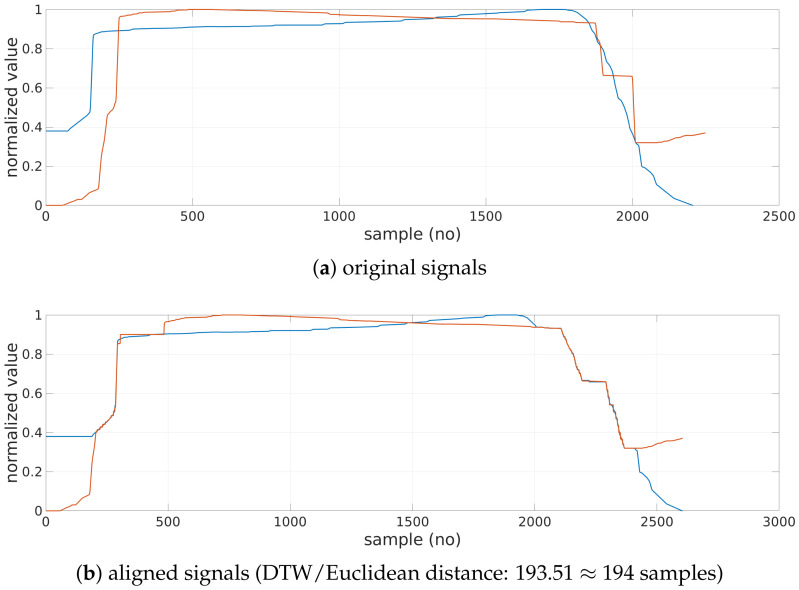
Model (orange) and examined (blue) characters (**a**) before DTW and (**b**) after DTW.

Once the models are selected, shape recognition is performed for all the patient’s characters not included in the model set (Figure 6). DTW is computed, and the nearest model (one of the triangles or one of the rectangles) determines the shape of the examined character.

### 2.5. Feature Extraction

Features are extracted from both the image and the signal form of the LAST. The characters (defined as sub-images or continuous samples in signal LAST) are analyzed both directly and after normalization. For normalization, each spatial dimension is standardized to the first (template) sign written by the examiner (i.e., first triangle or first rectangle in the sequence).

In the image feature extraction step, the dimensions of the characters are regarded in two ways: (1) as the width and height of the character’s bounding box or (2) as the width and height of the characters subjected to the ”straightening procedure” in which skewed or rotated characters are aligned based on the orientation of the ellipse defined as having the same normalized second central moment as the character (the inclination of the longer axis of the ellipse determines the rotation angle).

The full list of character-related features is included in the Appendix A.

#### NW Coefficient

**Figure 7:**
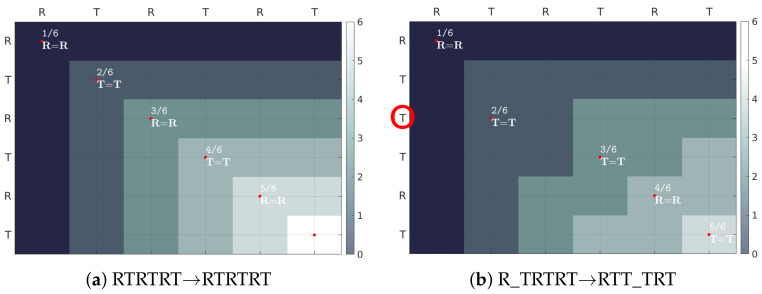
NW coefficient for two series of 6 characters: (**a**) identical/correct, NW = 100%, (**b**) incorrect NW = $\frac{5}{6}\approx$ 83%. Parameters: match bonus = 1, mismatch/gap penalty =0.

The evaluation of the entire sequence as a whole employs a novel index—the NW coefficient (Figure 7).

It is based on the Needleman–Wunsch algorithm [38] used in bioinformatics to assess the similarity between two protein or nucleotide sequences. The evaluated sequence *E* of length *n* is aligned with the standard (correct) sequence *G* of the same length. The scoring matrix *H* is calculated iteratively, starting with H(0,0)=0, according to the equation:

(2) $$\begin{array}{c} H_{EG}\left(i,j\right)=\max\left\{\begin{array}{cc} H_{i-1,j-1}+m+p & \mathrm{if}i,j\geq 1\\ H_{i-1,j}+g & \mathrm{if}i\geq 1\\ H_{i,j-1}+g & \mathrm{if}j\geq 1 \end{array}\right. \end{array},$$

for each cell (i,j), where *m* is the reward for a match and *p* is penalty for mismatch between characters G(i) and E(j), whereas *g* is the penalty for inserting a gap/deleting an element. In this paper, the reward *m* is set to 1 and the penalties *p* and *g* are set to 0. The normalization of the NW index (3) makes it independent of the sequence length.

(3) $$NW=\frac{\max\left(H_{EG}\right)}{\max\left(H_{GG}\right)}\cdot 100\%.$$

### 2.6. Classification

A proof of concept classifier is built. The Error Correcting Output Code [39] (ECOC) model is used to reduce a three-class classification task to a set of binary, Medium Gaussian Support Vector Machine (SVM) classifications [40,41]. The applied coding scheme implements a one-vs-one comparison strategy [42].

In the ECOC approach, implementing a one-vs-one classification strategy approach, the problem of classification into k-classes is reduced to k·(k−1)/2 binary classifications (e.g., in the paper, the classification into the three classes of PD, PSP, and control is performed by three binary classifiers–dichotomizers). Each of the dichotomizers learns one class vs. one-other-selected class and ignores the remaining ones. During prediction, the answers from all the dichotomizers give a codeword, which is matched based on the shortest Hamming distance to a codeword assigned to a final class.

In this paper, three Medium Gaussian Support Vector Machine (SVM) classifiers are used as dichotomizers. The Gaussian kernel function is employed:

(4) $$k\left(x_{i},x_{j}\right)=\exp\left(-\gamma |x_{i}-x_{j}{|}^{2}\right),$$

with γ set to $\sqrt{n}$, where *n* is the number of considered features.

During the proof of concept classification, only the statistically significant features (see Table A3, Table A4 and Table A5 in Appendix B) are selected.

## 3. Experiments and Results

The patient cohort consisted of three groups:

- PSP: 22 participants—11 women and 11 men (65 ± 8 years, diagnosed according to Litvan et al.’s criteria [43]);
- PD: 51 participants—19 women and 32 men (69 ± 7 years, diagnosed according to UK Brain Bank diagnostic criteria [44]);
- CON: 32 participants—18 women and 14 men (66 ± 8 years).

Three experiments were conducted: first, the correctness of the character separation step was assessed. Then, the shape recognition step was evaluated. Finally, the introduced features were examined to select a representative set of the patient’s neuropsychological state.

### 3.1. Character Separation

The evaluation of the automatic ROI selection was related to the manual delineations performed by an expert. The comparison was performed pixel-wise, based on the preprocessed image as described in Section 2.2. The evaluation was acceptable if a Jaccard similarity index [45] exceeded 70% (value selected experimentally by the expert performing manual delineations, on the basis of presented segmentation samples ordered by decreasing similarity).

Next, based on the number of acceptably/unacceptably separated characters, the Sørensen–Dice coefficient (DICE) for ROI was calculated, reaching 77.35 ± 15.30% in the PSP group, 79.32 ± 16.91% in the PD group, and 91.32 ± 10.27% in the control group.

### 3.2. Shape Recognition

Based on the automatically separated ROIs, shape recognition was performed. The results were assessed separately for rectangles (R) and triangles (T) using the Sørensen–Dice (DICE) coefficient. A reference labeling was obtained through the manual processing of the series by an expert (shapes were separated and individually assessed). Each shape correctly recognized was considered a true positive, whereas entities not recognized as analyzed characters (superfluously recognized as analyzed characters) were considered false negatives (false positives). Results are summarized in Table 1.

### 3.3. Feature Extraction

The feature extraction evaluation starts with the statistical assessment of the relationship between shape parameters and neuropsychological state.

Due to the imbalanced number of patients in each group, the Kruskal–Wallis ANOVA test was first applied to compare the groups (α=0.05). The parameters included in the ANOVA were the median in the groups and the interquartile range (differences between the third and first quartile, abbreviated IQR). The ANOVA was followed by the Dunn’s Multiple Comparison Test (significance level α=0.05) to determine in which group the mean ranks significantly differed from others.

The results are presented separately for the rectangles, triangles, and the NW coefficient. The latter reflects the entire sequence.

#### 3.3.1. Rectangles

As many as 28 of the 35 features proposed in the paper were significantly different between groups (*p* < 0.05) for rectangles (Table A3 in Appendix B). No single feature was significantly different between all groups. However, spatial-related parameters (top half of the table) were in most cases significantly different in PD or PSP patients, and the remaining two groups considered together. The largest width (MED Width, MED Width*) and height (MED Height, MED Height*), area (MED Area), dimensions of the ellipse describing the sign (MED Long axis, MED Short axis), convex hull area (MED Convex hull), and also signal length (MED Signal length) were calculated in the control and PSP groups. Lower values, but also with smaller interquartile range, were noted for the group with Parkinson’s disease.

The DTW distance from the artificially generated model (DTW model) was the smallest in the control group. This seems intuitive, as healthy patients should have the slightest difficulty in drawing characters closest to the model. The ratio of the sum of the widths of all the rectangles to the whole length of the series (Width ratio) was the largest in the PSP group. In the case of the control group, it was the closest to the desired value of 50% (indicating that the patient was able to save a similar amount of space on a sheet of paper for both types of characters).

#### 3.3.2. Triangles

In the triangles, 22 out of the 35 proposed features (Table A4 in Appendix B) were statistically different between groups (*p* < 0.05). Similarly as for the rectangles, no single feature was different between all considered groups, and spatial-related parameters (top half of the table) were more often than not different in at least one of the groups. A greater width (MED Width, MED Width*) and height before the straightening procedure (MED Height), area (MED Area), as well as the longer axis of the circumscribed ellipse (Long axis) and the convex hull area (Convex hull) were all calculated in the CON and PSP groups. Lower median and variability (in the form of standard deviation values and IQR) were obtained in the PD group. Interestingly, MED Height* (the median height of the triangles) and MED Short axis (the median length of the shorter ellipse axis) turned out to be significantly smaller in this group of patients. The orientation (angle of the ellipse, MED Angle) was different between the groups.

The DTW distance from the artificially generated model (DTW model) was the smallest in the control group—just as for rectangles. The ratio of the sum of the width of all triangles to the total length of the series (Width ratio) was the largest (and close to the optimum value of 50%) in the control group (47.49%).

#### 3.3.3. NW Coefficient

The value of the NW Coefficient introduced in the paper was statistically different between control and remaining groups (Table A5 in Appendix B). In the control group, the median of the results was 100% (i.e., perfect series), while in the PD and PSP group, it was 94.74% and 96.55%, respectively.

### 3.4. Performance

The method was implemented in the MATLAB R2018b (Linux) and run on a dual Intel Xeon E5-2630 processor (128GB RAM) equipped with NVIDIA GTX 1060 (3GB RAM). The total processing time (mean from three runs of fully automatic preprocessing, character separation, recognition, and feature extraction; all 105 cases) was 63,354 s (ca. 10 min per case). The preprocessing step contributed over 60% of this time. The implementation was not optimized for performance.

### 3.5. Classifiers

In order to evaluate the differentiating potential of the proposed features, an SVM classifier was constructed as described in Section 2.6. The five-fold cross-validation of the classification into three groups was based on features extracted using both expert (ground truth) delineations and automatic delineations. For the features extracted from expert delineations, accuracy when rectangle-only or triangle-only features were considered was 61.9% and 66.7%, respectively (Table 2). When the combined set of features was included, the observed accuracy was 69.5%. The addition of the NW coefficient improved the results in all cases, yielding the highest accuracy of 70.5% for the combined case.

For the same features extracted automatically, the observed accuracy for rectangle and triangle feature-based classifiers was 62.9% and 61.0%, respectively. The highest accuracy was noted when the NW coefficient was combined with rectangle and triangle-based features (66.7%).

## 4. Discussion

**Figure 8:**
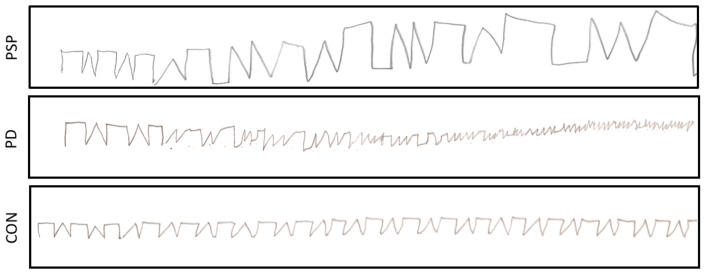
LAST execution examples in three different groups: Parkinson’s disease (PD), progressive supranuclear palsy (PSP), and neurologically intact (CON).

The accuracy of the automatic ROI delineation (shape separation) with average DICE index values over 75% is considered good. However, the results were the closest to the manual segmentation in the control group, in which the distinction between characters was clear, and worse results were observed for the more demanding cases (e.g., Figure 8). Similarly, the best automatic shape recognition (avg. DICE close to 90%) was observed in the control group, while worse results were received in the PSP or PD groups. However, the sequences consisting of malformed shapes were noted to be challenging for the expert as well.

The highest diagnostic accuracy of the presented methodology (approximately 67%) is lower than the automatic diagnosis based on volumetric magnetic resonance imaging, demonstrating an accuracy of above 90% [46,47,48,49,50]. However, this paper is the first attempt to use graphic features extracted from the paper LAST for automatic diagnostic classification, whereas neuroimaging indices requiring volumetric measurements have been developing for years. Moreover, the considered number of the features might be too high to classify the patient correctly, and other criteria than statistical significance alone should be considered for future work. However, the current group set-up is considered not well suited for the classification optimization purposes, as the groups are not evenly sized, and the number of cases is relatively small.

Nevertheless, it is worth pointing out that the Medium Gaussian SVM classification accuracy of the fully-automatic method (66.7%) is only slightly lower than the classification based on the manually-labeled data (70.5%), yet noticeably faster. Moreover, due to a low prevalence, the 22 person-group of the PSP patients is estimated to be nearly a 1% sample of the Polish population.

We hope that the proposed normalization of examined characters to the characters drawn by the specialist will allow for the comparison of LAST from different clinical sites: the normalized features should be less dependent on the individual traits, routines, and writing habits of the examiner.

From the neurological point of view, the impaired performance on LAST in PSP patients is likely to be multifactorial. It seems that the perseveration seen on LAST is a sensitive indicator of prefrontal and/or frontostriatal pathology. However, as perseveration is a sign of severe brain pathology, it may also be related to the degree of more widespread pathology rather than to discrete frontal lesions [51]. Moreover, oculomotor deficits in PSP patients may also influence the LAST outcome.

The results obtained using the introduced NW coefficient are highly promising. The obtained values are intuitive for interpretation (e.g., 100% for the perfect series in the control group) and statistically significant in evaluated groups. Moreover, employing the NW coefficient in the proof of concept classification increased the performance. However, it is worth pointing out that by setting the reward *m* to 1 and the penalty *p* to 0, the *NW coefficient* was reduced to a simple binary alignment. Nevertheless, the definition permits the *p* and *m* to be adjusted (as is common in nucleotide comparison in bioinformatics) with a psychiatrist’s observations in future studies. Moreover, the NW coefficient can be feasibly applied not only in computerized scenarios but also as an element of manual evaluation of the classic, paper–pencil LAST version (e.g., in clinical practice), complementing the usual, subjective approach. This suggests that the NW coefficient might become a standardized diagnostic tool for LAST evaluation.

## 5. Conclusions

In this study, an automatic feature extraction method was introduced. Based on automatically and manually generated character masks, standard and novel features describing the Luria’s Alternating Series Test (LAST) execution were extracted. The LAST is a widespread clinical tool in diagnosing neurodegenerative diseases, yet the assessment is not standardized, and its interpretation relies on the clinician’s expertise and remains qualitative. Furthermore, the LAST is mostly evaluated using only a ruler or a set square. Research involving new technologies such as digitizers is already conducted, yet its settings differ from clinical practice. In this study, all the usual restraints were preserved—the patient draws the shapes in front of a clinician using a pencil and paper sheet.

The digitization of the result enables us to measure not only standard character attributes such as character height or width, yet also its area, the dimensions of a circumscribed ellipse, and many more. The representation of the LAST as a signal, the estimation of the baseline using the BEADS algorithm, and the Dynamic Time Warping algorithm’s usage provided additional information about the drawn characters. These two combined approaches resulted in a feature vector consisting of 70 features, from which 50 turned out to be statistically significant (*p* < 0.05). A proof-of-concept Medium Gaussian SVM classifier built on selected features reached an accuracy of 69.5% in dividing the patient data into three groups: Parkinson’s disease (PD), progressive supranuclear palsy (PSP), and seniors with no neurological disorders (CON). To the best of our knowledge, such classification has not yet been performed elsewhere based on graphomotor features. Moreover, the addition of the novel *NW coefficient* improved the classification accuracy to 70.5%, making it a promising tool for sequence accuracy evaluation.

The implementation of the automatic division of the series into shapes, the character recognition, and automatic feature extraction presented in this paper is the first step towards the design of a fully automatic computer-aided diagnostic tool for neurodegenerative diseases. It is hoped that such a system might serve as a telemedical screening test for neurological disorders.

## Figures and Tables

**Table 1:** Results of the shape recognition approach.

Group	Shape	DICE [%]	STD
PSP	R	83.45	27.47
	T	81.13	28.67
PD	R	82.95	26.34
	T	86.76	25.18
CON	R	91.37	24.91
	T	89.24	24.65

**Table 2:** Classification accuracy [%] in three groups based on all shapes; in the bracket, the change of the value after adding the novel NW coefficient.

Method	Rectangles	Triangles	Both Shapes
Manually-labeled	61.9 (64.8)	66.7 (68.6)	69.5 (70.5)
Automatic	62.9 (65.7)	61.0 (61.9)	65.7 (66.7)

**Figure A1:**
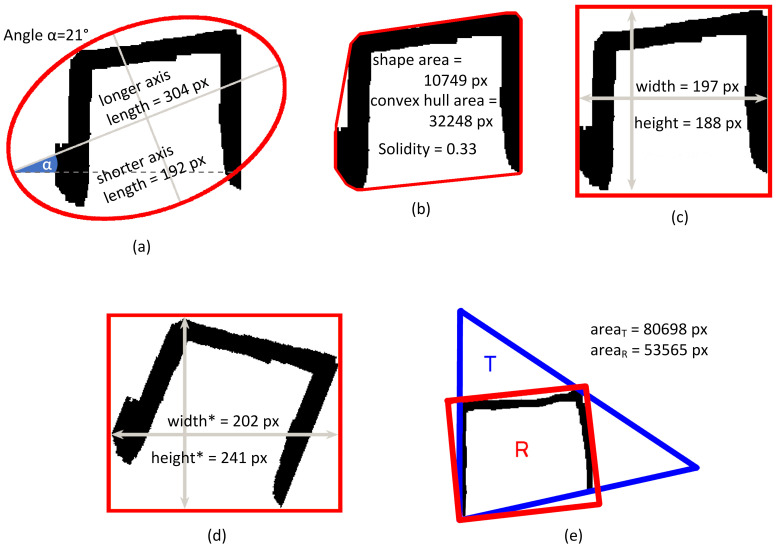
(**a**) ellipse with the same normalized second central moment as extracted character (rectangle); (**b**) character and its convex hull (red); (**c**) width and height of the character as width and height of the bounding box (red), (**d**) width and height of the character after rotation, (**e**) smallest triangle *T* and rectangle *R* circumscribing a character. In images lowercase-names mark features that will be normalized to the first (template) shape in series.

## Data Availability

The data presented in this study are available on request from the corresponding author.

## Appendix A. Luria’s Alternating Series Test Features

In this section, a full list of evaluated features is characterized. Selected image features have already been introduced in [32].

### Appendix A.1. Image-Based Features

The image feature vector for each shape group contains median values (MED) and standard deviations (STD) of all features of triangles/rectangles in the patient’s sequence.

Spatial and area-based features are normalized to the first shape of the same kind; i.e., to the template given by the examiner (e.g., the actual width of the rectangle as shown in the Figure A1c, in pixels, is divided by the width of the first template rectangle, in pixels, yielding normalized width with the dimensionless unit). All remaining features except *angle* (in degrees) are dimensionless as well. Features are defined in Table A1.

**Table A1:** Image-related features; features extracted after rotation are marked with *.

Name	Definition	Notes	Unit	Normalization
Width	The width of the bounding box of the character	See Figure A1c	–	Y
Height	The height of the bounding box of the character	See Figure A1c	–	Y
Area	The number of pixels of the character	Number of black pixels in the object in Figure A1b	–	Y
Convex hull	The number of pixels of the smallest convex polygon containing all the points of the character	See Figure A1b	–	Y
Solidity	The ratio of the pixels belonging to the character and the total number of pixels in the convex hull	Does not change after normalization; Solidity=Area/Convexhull See Figure A1b	–	N
Longer axis	The normalized length of the longer (major) axis of the ellipse having the same normalized second central moment as the character	See Figure A1a	–	Y
Shorter axis	The normalized length of the shorter (minor) axis of the ellipse having the same normalized second central moment as the character	See Figure A1a	–	Y
Angle	The inclination of the major axis of the ellipse having the same normalized second central moment as the character	See Figure A1a	[${}^{O}$]	N
Eccentricity	The measure of how much the ellipse deviates from being circular; [0,1]	$\frac{\sqrt{{\mathrm{Longer}\mathrm{axis}\mathrm{length}}^{2}+{\mathrm{Shorter}\mathrm{axis}\mathrm{length}}^{2}}}{\mathrm{Longer}\mathrm{axis}\mathrm{length}}$	–	N
Width${}^{*}$	The width of the bounding box the character after the rotation by the -Angle degrees (*straightening*)	See Figure A1d	–	Y
Height${}^{*}$	The height of the bounding box after the rotation by the -Angle degrees	See Figure A1d	–	Y
IF rectangle	The ratio of the area (interior pixels) of the smallest rectangle enclosing the character and the sum of the areas of the smallest rectangle and the smallest triangle circumscribing the character; the enclosing rectangle with the smallest area is found using Freeman approach [52]	$\frac{area_{R}}{area_{R}+area_{T}}$, where $area_{R}$ and $area_{T}$ denote quantities shown in Figure A1e; rectangle enclosing another rectangle should feature smaller area than enclosing triangle; as observed, regular triangles yield values close to 33%	–	N
IF triangle	The ratio of the area (interior pixels) of the triangle enclosing the character and the sum of the areas of the smallest rectangle and the smallest triangle enclosing the character; the enclosing triangle with the smallest area is found using O’Rourke approach [53]	$\frac{area_{T}}{area_{R}+area_{T}}$, where $area_{R}$ and $area_{T}$ denote quantities shown in Figure A1e; triangle enclosing another triangle should feature smaller area than the circumscribing rectangle; as observed, regular rectangles yield values close to 33%	–	N
Width ratio	The ratio of the sum of the widths of all characters representing one shape (rectangles or triangles) to the series’s whole length	Only patient-part of the series is considered	–	N

### Appendix A.2. Signal-Based Features

The signal feature vector for each shape group (rectangles or triangles) contains median values (MED) and standard deviations (STD) of the features defined in Table A2.

**Table A2:** Signal-related features.

Name	Definition	Notes	Unit
Histogram	The ratio of the number of the samples with the amplitude higher than the 80% of the maximum value of the signal to the total number of samples	See Figure 5; |A|/|S|, where *C* is a set of all samples, and A={c∈C:amplitude(c)>0.8ϵ}, and ϵ is a maximum signal value; in the ideal rectangle, the amplitude of most samples is equal to the maximum signal value (hence Histogram parameter is close to 100%), whereas in the ideal triangle, the amplitude values are uniformly distributed (hence Histogram parameter value is close to 20%); 80%, as well as 66% and 33% thresholds employed in Section 2.4 were determined experimentally for ten randomly selected rectangles and triangles drawn by patients in the control group	–
Variability	The standard deviation of number samples in the ten bins of the histogram	See Figure 5; width of each bin is set to a 10th of maximum signal value; each sample is assigned to exactly one bin of histogram	–
DTW model	The Dynamic Time Warping distance from the artificial (perfect) rectangle/triangle model closest to the character	Euclidean distance to the nearest template shape selected during DTW-based character recognition procedure as described in Section 2.4	no of samples
Signal length	The number of signal samples representing the character normalized to the first template character length	First shape of matching class (rectangle or triangle) written by the examiner is used as a template for normalization of patient-drawn shapes	–

## Appendix B. Statistical Evaluation of Features

In this section, the outcomes of the combined Kruskal-Wallis ANOVA and Dunn’s Multiple Comparison Test are presented, separately for the rectangles, triangles, and the LAST series as a whole, in Table A3, Table A4 and Table A5 respectively.

**Table A3:** Results of the Kruskal–Wallis ANOVA for rectangles. Statistically significant values in bold (*p* < 0.05). Groups with mean ranks significantly different from others are marked in gray. MED and STD by the feature name denote median and standard deviations for all triangles/rectangles in the patient’s sequence. Features extracted after rotation (see definition) are marked with *.

Feature	*p*	CON		PD		PSP	
		MED	IQR	MED	IQR	MED	IQR
MED Width [%]	<**0.001**	107.29	29.43	88.31	28.77	113.05	70.07
STD Width [%]	0.078	15.00	7.18	14.41	10.39	19.64	11.01
MED Height [%]	<**0.001**	114.18	39.82	98.20	28.89	131.78	62.03
STD Height [%]	**0.038**	11.38	6.51	11.52	5.21	15.73	7.24
MED Area [%]	<**0.001**	131.46	73.81	65.49	38.43	121.45	111.03
STD Area [%]	**0.001**	22.62	15.02	17.32	8.05	27.29	29.17
MED Width * [%]	**0.001**	118.99	33.39	103.83	29.16	135.89	54.99
STD Width * [%]	**0.042**	16.33	5.11	15.06	7.37	21.14	14.83
MED Height * [%]	<**0.001**	115.50	38.04	84.78	33.63	124.99	56.91
STD Height * [%]	**0.008**	16.06	10.11	16.40	8.43	22.08	13.70
MED Long axis [%]	<**0.001**	102.34	30.89	84.60	25.98	117.63	57.44
STD Long axis [%]	**0.025**	12.63	6.53	13.69	4.66	18.53	9.70
MED Short axis [%]	<**0.001**	109.11	37.56	84.19	29.09	118.37	45.40
STD Short axis [%]	<**0.001**	13.09	7.76	13.75	8.46	23.61	7.72
MED Angle	0.068	10.27	19.99	22.22	29.99	14.74	54.93
STD Angle	0.057	16.10	26.57	23.02	23.14	26.27	28.42
MED Solidity [%]	**0.028**	114.43	59.77	91.72	51.68	73.43	52.88
STD Solidity [%]	0.557	21.03	10.78	22.59	16.90	19.60	24.96
MED Eccentricity	0.190	0.69	0.14	0.74	0.11	0.71	0.17
STD Eccentricity	0.113	0.11	0.06	0.12	0.06	0.14	0.07
MED Convex hull [%]	<**0.001**	109.82	77.79	74.16	38.46	135.68	144.07
STD Convex hull [%]	**0.002**	23.36	14.55	18.25	13.93	35.96	34.23
MED Histogram [%]	**0.001**	73.14	8.62	64.99	11.39	67.58	12.73
STD Histogram [%]	**0.005**	10.34	5.96	12.63	6.78	15.35	7.18
MED Variability [%]	**0.014**	43.07	6.72	45.43	6.60	42.36	10.44
STD Variability [%]	<**0.001**	7.43	2.72	9.95	3.96	9.97	4.26
MED DTW model	**0.001**	94.88	28.10	112.91	31.92	125.51	39.20
STD DTW model	0.120	69.64	25.86	77.19	38.22	81.98	30.07
MED Signal length [%]	<**0.001**	108.69	29.08	88.07	29.61	110.72	37.50
STD Signal length [%]	**0.024**	15.19	6.77	15.61	9.45	20.42	18.57
MED IF rectangle [%]	<**0.001**	59.61	2.90	57.08	5.59	58.41	4.79
STD IF rectangle [%]	**0.013**	2.85	1.38	3.43	2.75	3.81	1.21
MED IF triangle [%]	<**0.001**	40.39	2.90	42.92	5.59	41.59	4.79
STD IF triangle [%]	**0.013**	2.85	1.38	3.43	2.75	3.81	1.21
Width ratio [%]	**0.001**	52.51	5.44	56.13	13.07	58.98	7.54

**Table A4:** Results of the Kruskal–Wallis ANOVA for triangles. Statistically significant values in bold (*p* < 0.05). Groups with mean ranks significantly different from others are marked in gray. MED and STD by the feature name denote median and standard deviation for all triangles/rectangles in the patient’s sequence. Features extracted after rotation (see definition) are marked with *.

Feature	*p*	CON		PD		PSP	
		MED	IQR	MED	IQR	MED	IQR
MED Width [%]	<**0.001**	93.20	36.04	69.26	30.14	70.28	56.56
STD Width [%]	0.443	15.49	6.19	16.46	10.08	18.36	12.94
MED Height [%]	<**0.001**	111.90	33.91	85.53	30.74	110.82	58.44
STD Height [%]	<**0.001**	11.27	6.88	11.76	4.98	18.38	7.34
MED Area [%]	<**0.001**	102.16	59.33	48.61	33.09	74.46	79.46
STD Area [%]	**0.001**	19.98	14.73	13.04	8.47	22.78	31.16
MED Width * [%]	**0.002**	105.21	36.55	92.67	29.64	101.21	63.56
STD Width * [%]	**0.002**	12.94	5.69	12.98	7.81	19.43	11.17
MED Height * [%]	<**0.001**	94.00	37.87	67.12	26.59	63.97	41.15
STD Height * [%]	0.327	14.33	6.39	14.47	7.94	15.25	7.05
MED Long axis [%]	<**0.001**	83.81	29.50	68.18	23.17	82.94	39.59
STD Long axis [%]	**0.003**	10.98	3.93	11.53	7.07	16.48	10.94
MED Short axis [%]	<**0.001**	82.38	35.37	57.60	22.56	57.44	53.17
STD Short axis [%]	**0.012**	12.92	4.48	12.84	5.40	15.86	7.55
MED Angle	**0.002**	34.81	38.62	45.90	23.03	62.22	16.18
STD Angle	**0.045**	27.16	25.30	30.29	32.69	42.67	40.45
MED Solidity [%]	0.706	138.21	77.97	128.58	77.68	171.15	129.25
STD Solidity [%]	**0.004**	29.29	20.34	34.95	22.49	51.27	53.71
MED Eccentricity	<**0.001**	0.74	0.09	0.81	0.11	0.85	0.13
STD Eccentricity	0.813	0.11	0.05	0.11	0.05	0.11	0.06
MED Convex hull [%]	<**0.001**	62.55	52.56	39.52	22.20	50.91	66.62
STD Convex hull [%]	**0.034**	14.10	5.86	12.04	10.34	17.60	11.88
MED Histogram [%]	0.169	26.25	2.90	26.26	4.08	24.03	8.04
STD Histogram [%]	**0.023**	6.35	5.46	9.34	5.12	9.25	6.24
MED Variability [%]	0.056	66.01	3.33	63.87	5.78	64.53	3.44
STD Variability [%]	<**0.001**	7.84	2.89	9.40	5.02	10.64	5.90
MED DTW model	<**0.001**	101.30	35.54	124.08	42.38	167.62	78.85
STD DTW model	**0.004**	75.61	28.09	69.44	52.04	103.59	47.32
MED Signal length [%]	<**0.001**	86.04	40.74	65.86	25.03	59.16	50.94
STD Signal length [%]	0.815	16.30	7.21	16.34	11.32	17.69	9.83
MED IF rectangle [%]	0.343	38.16	1.88	38.92	2.64	38.98	2.41
STD IF rectangle [%]	0.074	1.75	0.99	2.11	2.16	2.29	1.63
MED IF triangle [%]	0.343	61.84	1.88	61.08	2.64	61.02	2.41
STD IF triangle [%]	0.074	1.75	0.99	2.11	2.16	2.29	1.63
Width ratio [%]	**0.001**	47.49	5.44	43.49	13.07	41.02	7.54

**Table A5:** Results of the Kruskal–Wallis ANOVA for the whole series. Statistically significant values in bold (*p* < 0.05). The group with mean ranks significantly different from others is marked in gray.

Feature	*p*	CON		PD		PSP	
		MED	IQR	MED	IQR	MED	IQR
NW coefficient [%]	<**0.001**	100.00	2.20	94.74	15.56	96.55	10.00
